# Species Extinction, Biodiversity, Human Health, and Inevitable Role of Lifestyle Medicine: A Narrative Review

**DOI:** 10.1177/15598276261464949

**Published:** 2026-07-01

**Authors:** John Stevens

**Affiliations:** 1 National Centre for Natural Medicine, Co Founder, the Australasian Society of Lifestyle Medicine, 4571 Southern Cross University, Lismore, NSW, Australia (JS)

**Keywords:** biodiversity loss, species extinction, lifestyle medicine, planetary health, ecosystem services, zoonotic diseases, One Health, microbiome, health co-benefits, sustainable healthcare

## Abstract

Human health and the health of our planet are inextricably linked. The accelerating loss of global biodiversity represents one of the most profound health threats of the 21st century. With species extinction rates estimated to be 10-100 times higher than natural baselines, biodiversity decline is no longer solely an environmental concern. This narrative review synthesizes evidence suggesting that biodiversity decline is increasingly relevant as a determinant of human health and survival rather than solely an environmental concern. The six pillars of lifestyle medicine, offer a coherent framework for interventions that can simultaneously prevent and improve lifestyle related illness outcomes while improving planetary health by reducing environmental pressures that drive species extinction and biodegradation. The review examines evidence synthesized from peer-reviewed databases (MEDLINE, PubMed, CINAHL, Joanna Briggs, SCOPUS, ScienceDirect, and GreenFILE), primarily 2010-2025, organized across seven thematic domains: infectious disease ecology, ecosystem services, microbiome dynamics, lifestyle medicine interventions, One Health integration, behavioural change, and clinical/policy implications. The review argues that lifestyle medicine must evolve from individual-focused clinical practice to also explicitly address structural drivers of ecological degradation, including food systems, transport, and urban design, thereby operationalizing planetary health principles in clinical care.

“A lifestyle medicine that remains silent on environmental implications will be increasingly misaligned with the realities of the Anthropocene”.

So far as we know, Earth is the only planet which supports life, and it is the only planet on which we can survive. Our bodies and our minds are fashioned by it. Our hearts resonate with it. There will be little joy for the human spirit if we destroy the natural fabric of Earth with nothing left to do but go shopping. When we imagine the world a century from now, when we look our great grandchildren in the eye and see them smiling back at us because they know we cared for them, we smile too!

Dr Bob Brown (medical practitioner, former Australian Federal Senator and now conservationist)^1^

## Introduction

This review examines the accelerating loss of global biodiversity as a fundamental determinant of human health and survival and situates lifestyle medicine within this emerging planetary health context. Drawing on major international assessments and recent empirical work, the review outlines the evidence that species extinction and ecosystem degradation are occurring at unprecedented rates and are eroding the ecological foundations of population health. It then positions the rise of non-communicable diseases and the development of lifestyle medicine as a convergent response to these intertwined crises. The evidence from the literature establishes the rationale for treating biodiversity loss not as a peripheral environmental concern but as a core upstream driver of disease, while simultaneously framing lifestyle medicine as a clinically grounded approach capable of addressing both human and planetary health.

Human health and the health of our planet are inextricably linked. Our civilization depends on human health, flourishing natural systems, and the wise stewardship of natural resources. The *Lancet One Health Commission (LOHC)*, launched on July 17, 2025, highlights the urgent need for coordinated efforts across human, animal, and environmental health.^1^

The living fabric of the planet is undergoing rapid, human-driven erosion. The 2019 Intergovernmental Science-Policy Platform on Biodiversity and Ecosystem Services (IPBES) Global Assessment^2^ concluded that approximately 1 million animal and plant species face extinction within decades, many within the lifetime of current generations. Current extinction rates are estimated to be tens to hundreds of times higher than background rates over the past 10 million years, prompting descriptions of a sixth mass extinction event. Unlike previous mass extinctions triggered by volcanic activity, meteor impacts, or natural climate shifts, this event is overwhelmingly caused by anthropogenic drivers: land-use change, overexploitation, climate change, pollution, and invasive species.^1^

The health sector has traditionally regarded biodiversity as an environmental concern rather than a core determinant of clinical outcomes. However, multiple high-level assessments including the Millennium Ecosystem Assessment, IPBES, and recent World Health Organization (WHO) reports^2,3^ have reframed biodiversity loss as a direct and indirect threat to human health.^1,2^ Ecosystems provide provisioning services (food, fresh water, medicines), regulating services (disease control, air and water purification, climate regulation), supporting services (soil formation, nutrient cycling), and cultural services (recreation, spiritual value, sense of place). As ecosystems are simplified and species are lost, these life-support functions are destabilized in ways that alter patterns of infectious disease,^4-6^ nutrition,^7^ chronic disease risk,^8^ and mental health.^9^

Modern health challenges sit firmly within this ecological context.^10^ Non-communicable diseases (NCDs) like cardiovascular disease, diabetes, cancer, and chronic respiratory disease account for over 70% of global deaths and are driven largely by modifiable lifestyle factors. Yet the same dietary patterns, physical inactivity, and consumption patterns that fuel NCDs are also key drivers of biodiversity loss and climate change.^10^ The global food system alone is responsible for more than one-third of anthropogenic greenhouse gas emissions and is the single largest driver of biodiversity loss.^1,2,7,11^ Transport and urban design shape both physical activity patterns and environmental impacts.^12^ Health care itself contributes approximately 4-5% of global greenhouse gas emissions, with additional impacts on air pollution and resource use.^13^

Lifestyle medicine, defined as the use of evidence-based lifestyle therapeutic approaches including a predominantly whole-food, plant-predominant diet, regular physical activity, adequate sleep, stress management, avoidance of risky substances, and positive social connections to prevent, treat, and often reverse chronic disease,^10^ is therefore uniquely positioned at the nexus of personal health and planetary health. A growing literature in planetary health and One Health (see Table 1) demonstrates that interventions in lifestyle domains can produce large health co-benefits while reducing environmental pressure on ecosystems.^1-3,10,14-17^

**Table 1.** Definition of Key Terms.

Term	Definition
Biodiversity	The variety and variability of life on Earth, including diversity within species (genetic), between species, and of ecosystems, as well as the ecological and evolutionary processes that support this diversity.
Ecosystem services	The benefits people obtain from ecosystems, including provisioning services (e.g. food, water, fibre), regulating services (e.g. climate regulation, disease control, pollination), cultural services (e.g. recreation, spiritual value, sense of place), and supporting services (e.g. soil formation, nutrient cycling) that maintain the conditions for life.
Extinction	The permanent disappearance of a species, subspecies, or other taxonomic unit from Earth, occurring when the last surviving individual dies and the lineage can no longer contribute genetically or functionally to ecosystems.
Lifestyle medicine	A medical discipline that uses evidence-based lifestyle therapeutic approaches, including a predominantly whole-food, plant-predominant eating pattern, regular physical activity, restorative sleep, stress management, avoidance of risky substances, and positive social connection, to prevent, treat, and often reverse chronic disease and promote overall health and wellbeing.
Medicinal biodiversity	The component of biodiversity that provides actual or potential medical value, encompassing species, genes, and ecosystems that yield pharmaceuticals, traditional medicines, bioactive compounds, and knowledge systems that support prevention, diagnosis, and treatment of disease.
One health	A collaborative, multisectoral, and transdisciplinary approach that recognizes the interconnection between the health of humans, animals, and ecosystems, and seeks to design and implement programs, policies, and research in which multiple sectors work together to achieve optimal health outcomes for all.
Planetary health	A field and framing that focuses on the health of human civilizations and the state of the natural systems on which they depend, emphasizing that human health, equity, and wellbeing are inseparable from the integrity, stability, and resilience of Earth’s biophysical systems and planetary boundaries.
Planetary syndemic	The synergistic interaction of multiple global epidemics, classically obesity, undernutrition, and climate change, that share common systemic drivers and amplify each other’s health, social, and environmental harms, creating a combined burden greater than the sum of the individual problems.
Zoonotic	Referring to diseases or infections that are naturally transmitted between vertebrate animals and humans, either directly (e.g. bites, contact, consumption) or indirectly through vectors, food, water, or environmental contamination.

## Methods

This literature review aimed to examine the evidence for the relationship between species extinction, biodiversity loss and human health and survival, and the role lifestyle medicine could play in this equation.

The search for evidence led to the use of library data bases that included; medline, pubmed, cinhal, Joanna Briggs, infoRMIT, GreenFILE, ScienceDirect, and SCOPUS, as well as key world organizations such as the United Nations and WHO. The search was undertaken between August and December 2025, the review attempting to accurately reflect what is known up to this point in time. A range of search terms were used with words or terms that encompassed or otherwise described; biodiversity loss or extinction, and/or planetary health, and human health and survival, and lifestyle medicine. Some examples of broad search strings included: S*pecies extinction and biodiversity loss and human health, Biodiversity loss human health and infectious disease, and zoonotic diseases*. *Biodiversity loss and human health and microbiome or nutrition, or physical activity or stress or mental health or sleep or substance misuse or social connection, One Health framework and or planetary health and lifestyle medicine or nutrition or physical activity or sleep, or stress or mental health or substance misuse or social connection.*

The review synthesized information from contemporary, peer-reviewed and English-only language publications extensively (the only exceptions were for relevant peak body organization reports such as the World Health Organisation, the Secretariat of the Convention on Biological Diversity, 2005 and the United Nations), mostly from the years 2010 to 2025 (with contemporaneous exceptions when foundational research and concepts were relevant).

The narrative is organized by seven themes emerging from the literature analysis which also offered a logical flow of ideas leading to the seventh and final heading: *Clinical and policy implications*. Thus the remainder of the article is structured under these seven main headings as follows: (1) biodiversity loss and emerging infectious disease; (2) ecosystem services and human survival; (3) extended mechanisms including microbiome diversity and environmental mental health; (4) the role of lifestyle medicine across all six pillars; (5) the One Health framework and integration with lifestyle medicine; (6) behavioural change and structural interventions; and (7) clinical and policy implications.

Throughout, health is conceptualized not as an isolated biomedical outcome but as emergent from nested social-ecological systems. The review argues that lifestyle medicine should evolve from a primarily individual-focused discipline to one that explicitly engages with structural drivers of ecological degradation, including food systems, transport, urban design, as well as health care’s own carbon footprint. The evidence presented in this review contends that aligning Lifestyle Medicine with planetary priorities is increasingly urgent within the practice of medicine for societies as they work to remain within planetary boundaries while safeguarding the biodiversity.

### Biodiversity Loss and Emerging Infectious Disease

This section reports on the literature linking biodiversity loss to the emergence and transmission of infectious diseases, with a particular focus on zoonotic pathogens. A growing body of ecological and epidemiological research indicates that changes in species richness, community composition, and habitat structure can alter host–pathogen dynamics in ways that increase spillover risk to humans. The review, therefore, examine the dilution effect, deforestation and land-use change, and antimicrobial resistance in intensive agriculture as interconnected mechanisms through which ecosystem disruption reshapes disease ecology. By organizing the subheadings around these mechanisms, the narrative aimed to demonstrate how specific dimensions of biodiversity loss converge to influence zoonotic risk and to clarify why preventing species extinction is inseparable from preventing future epidemics and pandemics.

#### The Dilution Effect and Disease Ecology

According to the Convention on Biological Diversity (2005),^18^ biodiversity is defined as:

*the variability among living organisms from all sources including,* inter alia*, terrestrial, marine and other aquatic ecosystems and the ecological complexes of which they are part; this includes diversity within species, between species and of ecosystems.*^18^

A central pathway linking biodiversity loss and human health is the alteration of infectious disease dynamics. More than 60% of emerging infectious diseases are zoonotic in origin, and over 70% of these originate in wildlife.^5,6,19^ Traditional narratives viewed high-biodiversity regions as dangerous pathogen reservoirs. However, accumulating evidence from ecology and epidemiology reveals a more nuanced picture: in many systems, biodiversity appears protective, reducing disease risk through the ‘dilution effect’.^5^

The dilution effect posits that higher species richness can reduce pathogen transmission by ‘diluting’ encounters between pathogens and highly competent reservoir hosts, increasing the proportion of hosts that are poor at transmitting infection.^5^ Empirical studies across Lyme disease, West Nile virus, hantavirus, and other systems suggest that when ecosystems lose species, generalist, fast-living species with high reproduction and rapid turnover tend to persist; these species, including certain rodents and passerine birds, are often the most competent reservoirs for zoonotic pathogens. Conversely, larger-bodied, slower-breeding species that may serve as poor hosts disappear first under hunting pressure, habitat loss, and fragmentation.^5,19-24^

Several mechanistic pathways underpin this effect. First, species differ in their host competence ability to maintain and transmit a pathogen and in their contact rates with vectors and humans. Second, diverse communities often include predators and competitors that reduce densities of competent hosts and vectors. Third, intact ecosystems maintain balanced predator-prey and host-parasite dynamics, whereas defaunation, fragmentation, and climate change can destabilize these relationships, leading to unexpected amplifications of disease.^5,25,26^

A 2020 synthesis concluded that biodiversity loss underlies the dilution effect in many, though not all, disease systems, with the strongest evidence where host competence is highly skewed among species and where human disturbance preferentially removes low-competence hosts. Importantly, the dilution effect is not universal; in some systems, increased diversity may introduce more competent hosts (an *amplification effect*). Nevertheless, the preponderance of evidence suggests that rapid biodiversity loss, combined with expanding human encroachment into wildlife habitats, has increased the risk of zoonotic spillover and epidemic emergence.^5,19,20,22,27-32^

#### Deforestation, Fragmentation, and Spillover Interfaces

Land-use change and deforestation are consistently identified as dominant drivers of disease emergence. Tropical forests hotspots of biodiversity and carbon storage are converted to croplands, pasture, and settlements, creating fragmented landscapes characterized by forest edges, roads, and human settlements that bring people, livestock, vectors, and wildlife into closer contact.^5,28,31^

A seminal study of *Mycobacterium Ulcerans*, the causative agent of Buruli ulcer, in West Africa illustrates the complex effects of partial ecosystem disruption.^30,33^ Moderate levels of deforestation and land-use change produced maximal pathogen abundance because food webs collapsed in ways that removed predators but retained generalist hosts and vectors. This non-linear relationship implies that transition zones not pristine nor fully urbanized landscapes may pose the greatest spillover risk^5,30,33,^

Similar patterns have been observed in forest-fragmented landscapes in Africa, where clearing for agriculture and logging has been linked to Ebola outbreaks and other haemorrhagic fevers. Bats, primates, and rodents adapt to fragmented habitats and often forage in agroforestry systems, increasing human exposure through hunting, consumption, and contamination of food or water.^30,33^ Habitat fragmentation also concentrates wildlife into smaller areas, increasing intra-species and inter-species pathogen transmission and facilitating viral adaptation to novel hosts.^5,34,35^

Land reversion and restoration also carry risk if not carefully managed. Recent analysis suggests that poorly planned reforestation that increases edge habitat or introduces high-risk species could theoretically increase spillover in some contexts, underscoring the need for One Health-informed restoration. Collectively, these findings emphasize that species extinction and ecosystem simplification are intertwined with the spatial reconfiguration of habitats, which in turn shapes disease risk landscapes.^1,34,35^

#### Agricultural Intensification, Animal Agriculture, and AMR

Industrial animal agriculture exemplifies the convergence of biodiversity loss, zoonotic risk, and antimicrobial resistance (AMR). Expansion of livestock production drives deforestation, grassland conversion, and waterway pollution, contributing substantially to biodiversity decline.^2,11,36,^ High-density confinement systems create crowded, stressful conditions that favour pathogen transmission and necessitate prophylactic and metaphylactic use of antimicrobials. This practice accelerates the evolution and dissemination of resistant organisms.^2,37-40^

Low- and middle-income countries (LMICs), where regulatory systems may be weaker and antibiotics more readily available without prescription, face particular challenges in managing AMR from animal agriculture, though high-income countries contribute significantly to the overall burden.^37-39^ Resistant organisms and genes move between animals, humans, and the environment through direct contact, food consumption, manure application, water contamination, and wildlife movement. Wildlife including migratory birds and bats can act as *flying bridges*, transporting resistant bacteria across continents.^37-41^

AMR complicates the treatment of both human and animal infections, undermining the efficacy of modern medicine and increasing morbidity, mortality, and health care costs. A Lancet commissioned study calculated that 4.95 million deaths could be attributed to AMR in 2019.^39^

As biodiversity declines and habitats are converted to monocultures and intensive livestock operations, ecological checks and balances on pathogen dynamics are eroded, and selective pressures for resistance intensify. Lifestyle medicine’s promotion of dietary shifts away from industrial animal products, therefore, has implications not only for cardiometabolic health and greenhouse gas emissions but also for AMR and biodiversity conservation.^2,11,37-39^

### Ecosystem Services and Human Survival

The literature that was reviewed extended beyond infectious disease to consider how biodiversity underpins the full spectrum of ecosystem services on which human survival and wellbeing depend. The ecosystem services framework highlights that species-rich systems provide food, clean water, medicinal resources, climate regulation, and cultural and psychological benefits essential to health. In this section, therefore the narrative included evidence on pollinator decline and food security, medicinal biodiversity and pharmaceutical discovery, climate regulation and carbon sequestration. The subheadings in this section are intentionally linked: each addresses a distinct category of ecosystem services, yet together they illustrate that species extinction threatens multiple, interdependent pathways through which ecosystems sustain human life and resilience.

#### Pollination, Food Security, and Nutrition

Pollinators, bees, butterflies, birds, bats, and other animals, play a critical role in the reproduction of three-quarters of global food crops, particularly fruits, vegetables, nuts, and seeds that supply essential vitamins, minerals, and phytonutrients.^42^ Agricultural intensification, pesticide use, habitat loss, parasite spread, and climate change have collectively contributed to alarming declines in pollinator abundance and diversity.^42-44^

The Lancet commissioned modelling found that pollinator deficits, food consumption, and health outcomes, estimated that insufficient pollination currently reduces global production of fruits, vegetables, and nuts by 3-5%, translating into approximately 427,000 excess deaths annually from heart disease, stroke, diabetes, and certain cancers due to reduced intake of these protective foods.^45^ Mortality impacts are particularly pronounced in middle and high-income countries with higher NCD burdens and diets already low in fruits and vegetables, while low-income countries experience disproportionate agricultural production and income losses that threaten food security.^45^

Beyond macronutrient supply, pollinator decline jeopardizes micronutrient security. Many pollinator-dependent crops are key sources of vitamin A, folate, calcium, and other micronutrients critical for immune function, growth, and development.^46^ Modelling has revealed that a 50% loss of pollinators would be associated with 700,000 additional annual deaths and 13.2 million DALYs.^46^ The Lancet Planetary Health Commission^47^ has emphasized that healthy diets rich in fruits, vegetables, legumes, and nuts depend on intact pollination systems, making biodiversity conservation a prerequisite for sustainable nutrition.^47^

Lifestyle medicine’s emphasis on whole-food, plant-predominant diets hinges on sustained access to diverse plant foods. Without pollinators and functional agroecosystems, dietary recommendations become aspirational rather than attainable, particularly for vulnerable populations facing the *global syndemic* (a synergy of epidemics) of over and under nutrition.^48,49^

The *global syndemic* conceptual framework brings together three health-related challenges that traditionally have been addressed separately: obesity (including high BMI and associated diet-related non-communicable diseases); undernutrition (including stunting, wasting, underweight, and micronutrient deficiencies) and climate change (due to its sweeping effects on human and planetary health). These three pandemics exacerbate one another and stem from the same fundamental causes, primarily within the food and transport systems and overconsumption.^48,49^

The drivers for the *syndemic* include upstream determinants that are part of the Lifestyle Medicine discourse and they include:

- Powerful commercial interests of multinational food corporations (often called ‘Big Food’ and ‘Big Soda’) that promote ultra-processed foods.
- Inadequate political leadership and governance, leading to ‘policy inertia’ in implementing effective prevention policies.
- Business models that prioritize profit over human and ecological health.^48,49^

Lifestyle medicine has a responsibility to advocate for both upstream and drivers to transform food systems and at the clinical and community interface to educate, counsel and change behaviours that will contribute to health of the individual and the planet.

#### Medicinal Biodiversity and Pharmaceutical Discovery

Biodiversity provides the molecular library that underpins much of modern pharmacology. Estimates suggest that 50-80% of modern drugs are derived from or inspired by natural products, with 74% of plant-derived drugs discovered through investigations of species used in traditional medicine. Classic examples include paclitaxel from Pacific yew trees for cancer treatment, artemisinin from Artemisia annua for malaria, and ACE inhibitors derived from Brazilian pit viper venom.^50,51^

As habitats are cleared and species disappear, the opportunities for discovering novel bioactive compounds diminish irreversibly.^52^ Many tropical species have never been taxonomically described, let alone screened for pharmacological potential. Extinction thus closes off entire evolutionary lineages and biochemical pathways before their potential benefits can be explored.^50,52,53^

Beyond drug discovery, medicinal biodiversity supports local and Indigenous health systems. An estimated 80% of the global population relies at least partly on traditional medicine, often based on wild or cultivated plants and fungi.^53^ Overharvesting, habitat loss, and climate change threaten these resources, with disproportionate impacts on communities that contribute least to global environmental degradation. Opportunities exist to align conservation with ethical and equitable benefit sharing, recognizing Indigenous knowledge holders as co-stewards and co-innovators.^53-57^

From a lifestyle medicine perspective, the erosion of medicinal biodiversity carries two implications. First, it reduces the future storehouse available to treat diseases that lifestyle interventions cannot fully prevent or reverse (e.g. certain cancers, complex infections). Second, it destabilizes traditional health systems and cultural practices that can complement lifestyle medicine, particularly when integrated respectfully in Indigenous and local contexts.

#### Climate Regulation, Carbon Sequestration, and Extreme Events

Biodiverse ecosystems, especially intact forests, wetlands, mangroves, seagrass meadows, and peatlands serve as critical carbon sinks, absorbing and storing large amounts of atmospheric CO_2_. Forests alone sequester an estimated 2.6 billion tonnes of CO_2_ annually, moderating climate change and buffering communities against extreme weather.^58^ The loss of these ecosystems both reduces sequestration capacity and releases stored carbon through burning or decay, creating powerful positive feedback loops that accelerate global warming.^2,3,58-60^

A detailed analysis of forests in the western United States identified irrecoverable carbon, carbon that, if lost, could not be recovered by mid-century that is disproportionately stored in older, biodiverse forests with high structural complexity and intact ecological processes.^59^ Protecting these forests could sequester 1485 Tt (Tera tonne = 10^18^ grams) of carbon by 2099, equivalent to several years of regional fossil fuel emissions, while simultaneously preserving habitat for numerous threatened and endangered species. Similar co-benefits have been demonstrated for forest-based climate mitigation projects globally, where well-designed interventions deliver health benefits via reduced air pollution, improved livelihoods, and enhanced ecosystem services.^59,60^

Climate change itself has cascading health impacts, including heat-related illness, vector-borne and water-borne disease expansion, food and water insecurity, mental health complications, and displacement.^61,62^ As temperatures rise and extreme events increase in frequency and intensity, health systems are strained, and lifestyle interventions become harder to implement, for example, outdoor physical activity becomes unsafe during heatwaves or wildfire smoke events.

Species extinction and ecosystem degradation thus operate both upstream and downstream of climate change, reinforcing the need to address biodiversity, climate, and health together rather than as isolated domains. Lifestyle medicine can align its clinical goals with climate and biodiversity objectives by continuing to promote high-impact behaviours such as plant-predominant diets, active transport, and reduced overconsumption.

### Extended Pathways: Microbiome Diversity and Environmental Mental Health

The literature also suggests additional, more subtle pathways through which biodiversity loss affects human health, notably via environmental microbiomes and mental health responses to environmental degradation. In this section, the evidence explains how reduced exposure to diverse environmental microbiota influences human microbiome composition, immune regulation, and susceptibility to inflammatory and allergic disease. There is a growing recognition of ecological grief, eco-anxiety, and nature deficit as mental health consequences of environmental decline. Bringing these subheadings together highlight how macro-level ecological changes are mirrored in micro-level biological and psychological processes, reinforcing the view that biodiversity loss permeates both somatic and mental health domains.

#### Environmental Microbiomes, the Human Microbiome, and Immune Regulation

Emerging research indicates that biodiversity loss extends into the microscopic realm, with consequences for immune development and chronic disease. The ‘Old Friends’ or ‘biodiversity’ hypothesis proposes that reduced exposure to diverse environmental microbes through urban living, reduced contact with soil and animals, and highly sanitized environments disrupts human microbiome development and contributes to immune dysregulation, allergic disease, and inflammatory conditions.^63^

Comparative studies between urban and rural children, and between those exposed to natural environments vs more sterile settings, have shown that greater environmental biodiversity correlates with more diverse and functionally robust skin and gut microbiomes, more favourable cytokine profiles, and lower prevalence of atopic disease.^64^ A notable intervention study that increased biodiversity in daycare yards by adding forest floor, sod, and planters found that children exposed to these enriched environments developed more diverse skin and gut microbiota, increased regulatory

T-cells, and higher levels of anti-inflammatory cytokines within 1 month compared with controls.^65^

Environmental microbiome diversity and stability also appear to confer resilience to pathogenic invasion. Degraded ecosystems, including intensively managed urban green spaces or monoculture agricultural systems, harbour less diverse microbial communities that may be less capable of buffering against pathogen establishment and spread.^65,66^

Lifestyle medicine, particularly in its preventive orientation during pregnancy, infancy, and early childhood, can incorporate environmental microbiome considerations by encouraging nature play, contact with soil and non-urban green spaces, reduced overuse of broad-spectrum antibiotics, and consumption of fibre-rich, minimally processed foods that nourish a diverse gut microbiota.

#### Mental Health, Eco-Anxiety, and Nature Deficit

Mental health represents another critical domain where species extinction and environmental degradation exert growing influence. Environmental decline climate change, deforestation, biodiversity loss, pollution has been associated with increased anxiety, depression, trauma.^67^ Complex emotions specifically linked to environmental decline are emerging as new areas for concern such as ecological grief and solastalgia.^68-70^

Conversely, contact with natural environments, particularly biodiverse green and blue spaces, is associated with lower stress, better mood, improved cognitive function, and reduced incidence of depression and anxiety.^67,69^ Neighbourhood green space has been linked with healthier diurnal cortisol patterns and reduced perceived stress, especially in socioeconomically deprived communities.^71^ A scoping review of greenspace interventions found consistent reductions in cortisol, perceived stress, and negative affect, with some evidence of improved cardiovascular and metabolic markers.^71-77^

The emerging practice of *nature prescribing* in which clinicians formally recommend time in natural environments as part of treatment plans leverages these benefits.^78,79^ Green Social Prescribing programs in the United Kingdom and elsewhere connect patients to guided nature walks, community gardening, conservation volunteering, and outdoor group activities, reporting improvements in mental health, social connectedness, and physical activity. These programs highlight how biodiversity and ecosystem access function as therapeutic infrastructure. Environmental changes that eliminate or degrade natural spaces such as urban sprawl, deforestation, and species extinction, therefore, remove not only ecosystem services but also critical mental health resources.

For Indigenous peoples, whose cultural identity, spirituality, and knowledge systems are deeply entwined with specific species and landscapes, biodiversity loss can be experienced as cultural and spiritual injury, compounding the mental health burden of colonization and dispossession.^1,53,56,57^

Lifestyle medicine’s stress management and social connection pillars can explicitly incorporate nature-based practices, eco-therapeutic approaches, and community green initiatives, acknowledging that mental health is co-produced by social and ecological environments^80^

### The Role of Lifestyle Medicine in Addressing the Biodiversity Crisis

Having established the principal pathways linking biodiversity loss to human health, a main aim of this review was to consider the specific role that lifestyle medicine can and will inevitably play in responding to this crisis. Lifestyle medicine targets modifiable behaviours that are simultaneously major drivers of chronic disease and significant contributors to environmental degradation. This section, therefore, examines how whole-food, plant-predominant nutrition, physical activity and active transport, restorative sleep, stress management (including nature-based interventions), social connection, and avoidance of risky substances each intersect with biodiversity-relevant processes. The subheadings are organized around the six pillars of lifestyle medicine, with each pillar mapped to corresponding ecological impacts, to show that lifestyle prescriptions can function as deliberate strategies for both chronic disease prevention and biodiversity conservation.

#### Whole-Food, Plant-Predominant Nutrition as an Ecological Intervention

The global food system is the single largest driver of biodiversity loss, responsible for roughly 70-85% of terrestrial biodiversity threats through land-use change, pesticide use, water extraction, and nutrient pollution. Ruminant livestock and animal feed production account for a disproportionate share of agricultural land, greenhouse gas emissions, and habitat conversion.^7,11,45,46,48^

The EAT-Lancet Commission’s planetary health diet outlines a dietary pattern that can sustain 10 billion people within planetary boundaries while reducing diet-related NCDs.^7^ This pattern emphasizes whole grains, legumes, fruits, vegetables, nuts, and unsaturated oils; includes modest amounts of dairy, poultry, and fish; and minimizes red meat, processed meat, and added sugars. Modelling suggests that widespread adoption could prevent 11-15 million deaths annually and reduce diet-related greenhouse gas emissions by up to 70% by 2050, while freeing large areas of land for conservation and restoration.^7,11^

Estimates indicate that transitioning from a typical Western diet to a vegan diet could reduce global agricultural land use by up to 75%, largely by eliminating pasture and reducing cropland devoted to animal feed.^7,11^ In addition, it has been shown that GHG emissions in meat-eaters are approximately twice as high as those in vegans. It is likely that reductions in meat consumption would lead to reductions in dietary GHG emissions as well.^81^

Even partial shifts such as reducing red meat consumption by 50%, yield substantial reductions in land use, water use, and emissions.^11,31,81,82^ These reductions directly translate into lower pressure on forests, grasslands, and freshwater systems, creating space for biodiversity recovery.^7,11,82^

Lifestyle medicine practitioners routinely counsel patients on plant-predominant diets to prevent and manage NCDs. Integrating biodiversity and climate considerations into these consultations can strengthen motivation by highlighting health co-benefits and ethical dimensions. For example, framing a transition to a plant-predominant diet to treat both coronary artery disease and planetary degradation could resonate with patients' values, particularly when accompanied by specific, culturally appropriate strategies.^14,15,80^

At the same time, clinicians must be attentive to equity and the *syndemic* of malnutrition, in which undernutrition, micronutrient deficiencies, and obesity coexist in the same populations or individuals.^48^

Recommendations should be adapted to local contexts, considering affordability, accessibility, cultural foodways, and Indigenous food sovereignty. Collaboration and co-design with public health and policy actors is necessary to align dietary guidance with structural reforms in agricultural subsidies, food environments, and marketing regulations.^7,10,11,48^

#### Physical Activity, Active Transport, and Urban Design

Physical inactivity is a leading risk factor for cardiovascular disease, diabetes, certain cancers, and premature mortality.^83^ Simultaneously, transport is a major contributor to greenhouse gas emissions and urban air pollution, with road traffic also degrading biodiversity through habitat fragmentation, roadkill, and noise pollution.^1,8,10,18^

Lifestyle medicine encourages regular moderate-to-vigorous physical activity, which can be synergistically achieved through active transport, walking, cycling, wheelchair use, and public transport.^8^ WHO Europe highlights that increasing active mobility not only reduces physical inactivity but also lowers air pollution, noise, greenhouse gas emissions, and traffic injury risk. A shift toward active transport has been described as a *no-regrets* strategy because it delivers benefits under virtually all plausible futures.^83,84^

Sustainable physical activity programs that promote walking and cycling, particularly when integrated into daily routines (e.g. commuting, school travel), have been shown to reduce carbon footprints per participant while improving cardiometabolic health.^83^ However, the feasibility of outdoor activity is threatened by extreme heat, wildfires, and poor air quality,^85^ especially in disadvantaged neighbourhoods with limited tree cover and green infrastructure.^86^

Lifestyle medicine must therefore move beyond individual-level exercise prescriptions to advocate for urban designs that create safe, accessible, biodiverse environments for physical activity. This includes protected bike lanes, walkable neighbourhoods, green corridors, and urban tree canopies that reduce urban heat islands and provide habitat as suggested by WHO guidelines.^83^ Such environments support both human and non-human species, aligning physical activity promotion with habitat connectivity and biodiversity goals.^77,84^

#### Stress Management, Nature Exposure, and Microbiome-Supporting Practices

Stress management is a core pillar of lifestyle medicine, with chronic stress recognized as a driver of cardiometabolic disease, mental health disorders, and immune dysfunction.^87,88^ Nature-based interventions including forest bathing, green exercise, gardening, and conservation volunteering offer evidence-based tools for stress reduction, with additional benefits for microbiome diversity, physical activity, and social connection.^71-74,78-80,84^

The concept of ‘nature as medicine’ has been advanced as a potential seventh pillar of lifestyle medicine.^80^ Mechanisms for its benefits include modulation of the hypothalamic–pituitary–adrenal axis and cortisol patterns, increases in parasympathetic activity, exposure to phytoncides and other bioactive plant compounds, enhancement of gut and skin microbiome diversity, and provision of restorative environments that reduce cognitive load.^71,74,75,80^

Practically, clinicians can prescribe specific doses of nature exposure (e.g. 120 minutes per week, with at least 20-30 minutes per session) in parks, forests, waterways, or community gardens. These prescriptions can be embedded within management plans for hypertension, depression, anxiety, pain, and sleep disorders, among others. Collaboration with local governments, parks agencies, and community organizations can facilitate structured programs and reduce barriers, particularly for people in nature-poor environments. From a biodiversity perspective, the evidence suggests that nature prescriptions are most effective when they involve biodiverse environments rather than low-diversity lawns or heavily manicured spaces.

This reinforces the need for urban biodiversity planning and conservation of peri-urban and rural natural areas. It also underscores that environmental degradation is a direct threat to an emerging category of low-cost, high-impact non-pharmacological therapies.^77,86^

#### Sleep, Environmental Quality, and Circadian Health

Sleep health is a pillar of lifestyle medicine, with inadequate sleep linked to obesity, diabetes, cardiovascular disease, and mental health disorders.^87,89^ Environmental conditions, including air pollution, noise, light pollution, and ambient temperature, profoundly affect sleep onset, duration, and quality.

Sleep disturbance serves as a potent activator of systemic inflammation, with substantial implications for chronic disease pathogenesis. A landmark systematic review and meta-analysis examining nearly 35,000 participants for C-reactive protein (CRP) and over 3000 participants for interleukin-6 (IL-6) demonstrated consistent associations between sleep disturbance and elevated inflammatory biomarkers.^90^ Sleep disturbance was associated with higher CRP levels (effect size 0.12, 95% CI 0.05-0.19) and elevated IL-6 across diverse populations. Importantly, both short sleep duration (typically <6 hours) and long sleep duration (typically >9 hours) exhibited associations with inflammation markers.^90^

Systematic reviews indicate that chronic exposure to fine particulate matter (PM^2.5^), NO_2_, and O_3_ is associated with shorter sleep duration, reduced sleep efficiency, sleep-disordered breathing, and increased insomnia symptoms.^91-93^ Mechanisms may involve airway inflammation and obstruction, central nervous system effects, altered melatonin secretion, and cardiovascular strain. Heatwaves further disrupt sleep, especially in urban areas with limited vegetation.^91-93^

As noted previously, biodiverse green spaces mitigate some of these stressors by filtering air pollutants, reducing noise, moderating urban temperatures, and providing darker nighttime environments. Therefore, environmental restoration and biodiversity-friendly urban design support sleep health, while lifestyle medicine can incorporate environmental assessments into sleep counselling, including discussions on air quality, bedroom environment, and timing of outdoor activity.

#### Social Connection, Community Resilience, and Environmental Stewardship

Social connection is another pillar of lifestyle medicine as it is a significant determinant of health. Social connection can also be a facilitator of collective environmental action. Communities with strong social capital-dense networks, trust, shared norms, are more resilient to climate-related and ecological shocks, including floods, heatwaves, wildfires, and pandemics. Socially cohesive communities have been shown to better coordinate emergency responses, distribute resources, and advocate for protective policies.^94-98^

Lifestyle medicine increasingly recognizes loneliness and social isolation as risk factors comparable to traditional cardiometabolic risk factors.^99,100^ Loneliness and social stress have been shown to be associated with activation of the hypothalamic-pituitary-adrenocortical axis and the sympathetic nervous system. There is evidence to suggest that chronic social stress leads to glucocorticoid resistance, enhanced myelopoiesis, upregulated proinflammatory gene expression, and oxidative stress.^99^ The mechanisms in the development of loneliness-associated CVD remains unclear.

Interventions such as group-based programs, shared medical appointments, and community-based lifestyle interventions not only improve health behaviours but also build social networks.^79,101^

When these interventions are nature-based, for example, group walks in parks, community gardening, or conservation volunteering, they simultaneously promote environmental stewardship and biodiversity awareness.^79,94,96,97^

Green social prescribing schemes in the UK and elsewhere demonstrate that connecting individuals with nature-based group activities can reduce loneliness, improve mental health, increase physical activity, and strengthen community resilience.^102,103^ These programs align naturally with lifestyle medicine's social connection pillar and represent a model for integrating healthcare, public health, and environmental agencies in a One Health framework.

#### Avoidance of Risky Substances and Environmental Toxins

Lifestyle medicine’s avoidance pillar traditionally focuses on tobacco, alcohol, and other addictive substances, as well as ultra-processed foods. Expanding this pillar to encompass environmental toxins including pesticides, endocrine-disrupting chemicals, heavy metals, and air pollutants, reflects a broader understanding of risky substances that undermine both human and ecological health.^1,10,47^

Environmental toxins can elicit oxidative stress, a condition characterized by an imbalance between the production of reactive oxygen species (ROS) and the body’s ability to detoxify and repair the resulting damage. Oxidative stress triggers a cascade of events, including inflammation, endothelial dysfunction, lipid peroxidation, and vascular remodelling, which can contribute to the development of atherosclerosis, hypertension, and other cardiovascular and related pathologies for example.^104,105^

As previously reported above, pesticide use reduces biodiversity including pollinators, soil biota, and aquatic life, and has also been associated with increased risks of cancer, neurological disease, developmental disorders, and endocrine disruption in humans. Air pollutants contribute to NCDs while also harming vegetation and wildlife. Lifestyle medicine practitioners can collaborate with patients about reducing personal exposure (e.g. smoking cessation, indoor air quality, avoiding some household chemicals) while advocating for upstream regulatory and policy changes that protect both people and ecosystems.

### The One Health Framework and Lifestyle Medicine Integration

In this section the review considers how lifestyle medicine competencies, particularly prevention and behaviour change, can complement and operationalize the One Health agenda. The narrative argues that lifestyle medicine should be understood not as a parallel endeavour but as a clinical expression of One Health, capable of translating high-level frameworks into concrete interventions at the level of patients, families, and communities.

The One Health approach recognizes the interdependence of human, animal, and environmental health and calls for multisectoral, transdisciplinary collaboration to address shared threats. National and international frameworks increasingly adopt One Health principles to manage zoonotic diseases, AMR, and environmental risks.^1,106^

Lifestyle medicine naturally aligns with One Health through its upstream focus on behaviour and environmental determinants.^10,80^ As the evidence presented in this review suggests, for example, reducing consumption of industrially produced animal products addresses NCDs, mitigates AMR risk, and reduces pressure on natural habitats. Encouraging active transport reduces cardiovascular risk, injuries from sedentarism, and emissions that harm both human and wildlife health. Nature-based interventions promote mental health while building support for conservation policies.

Integrating One Health explicitly into lifestyle medicine practice would involve several steps:

- Including planetary health and One Health content in lifestyle medicine training and certification.
- Developing clinical tools to assess patients' environmental exposures, nature access, and environmental distress.
- Encouraging clinicians to participate in intersectoral collaborations with veterinary, environmental, urban planning, and agricultural partners.
- Incorporating One Health metrics (e.g. reduced meat consumption, reduced car use, increased nature contact) as secondary outcomes in lifestyle medicine interventions.

These few examples of integration, positions lifestyle medicine as a clinical expression of planetary health and One Health; an applied discipline that operationalizes planetary boundaries and biodiversity conservation within everyday practice.

### Behavioural Change and Structural Interventions for Sustainable Health

These next 2 sections examine the insights developed from the evidence in the preceding sections and how these might be translated into practice, systems change, and policy. While lifestyle medicine has traditionally focused on individual behaviour change, the planetary health and biodiversity literature consistently highlights the importance of structural and policy interventions in shaping what is possible for individuals. The review examines behaviour change techniques for climate, and biodiversity-friendly lifestyles including the opportunity to learn from Indigenous knowledge and with First Nations communities, the role of health systems in reducing their own ecological footprint.

Lifestyle medicine has traditionally focused on individual behaviour change, often using motivational interviewing, goal setting, and self-monitoring. However, behaviours are embedded within social, economic, and environmental structures that can either facilitate or obstruct change. Addressing species extinction and planetary health requires lifestyle medicine to engage at multiple levels: individual, community, organizational, and policy.^107-109^

Behaviour change research identifies several effective techniques for promoting sustainable behaviours, including information provision, feedback, goal setting, social comparison, and environmental restructuring. For diet, interventions that combine health and environmental messaging, provide practical skills (cooking, shopping), and modify food environments (cafeteria layouts, procurement policies) have been more successful than information-only approaches. For active transport, infrastructure changes (bike lanes, traffic calming, public transport improvements) are critical alongside individual counselling.^109^

Health care organizations themselves can serve as role models by reducing their ecological footprint through sustainable procurement, energy efficiency, waste reduction, and low-carbon models of care. Primary care carbon footprint analyses indicate that a significant share of emissions derives from pharmaceuticals, diagnostics, and patient and staff travel, suggesting opportunities for telehealth, deprescribing, and preventive care.^110-112^

Embedding lifestyle medicine within sustainable health system transformation amplifies impact. This results in preventing disease, decreases resource-intensive care, while lifestyle changes reduce external environmental pressures.

Finally, as previously presented, Indigenous knowledge systems offer critical insights into sustainable interactions with biodiversity, relational worldviews, and land stewardship practices that have sustained high levels of biodiversity for millennia. Integrating Indigenous and local knowledge with scientific approaches when undertaken with respect, consent, and fair benefit sharing can improve biodiversity governance and health outcomes. Lifestyle medicine clinicians can learn from these knowledge systems in re-embedding and framing health within ecological and cultural contexts.

### Clinical and Policy Implications

A distillation of the evidence offers clear guides for clinical practice and policy development.

According to the evidence presented above, clinically, lifestyle medicine practitioners should:

- Explicitly discuss environmental co-benefits of lifestyle interventions, framing plant-predominant diets, active transport, and nature exposure as strategies that benefit patients and the planet.
- Screen for environmental determinants of health (air quality, access to green space, food environments) and ecological distress (eco-anxiety, ecological grief).
- Prescribe nature exposure and socially connected, nature-based activities where feasible.
- Engage in shared decision-making that incorporates patients' environmental values.

At system and policy levels, lifestyle medicine organizations and clinicians can:

- Advocate for food system policies that support plant-predominant, minimally processed diets, including agricultural subsidies, public procurement standards, and marketing regulations.
- Support urban planning and transport policies that prioritize active transport, green infrastructure, and biodiversity-friendly designs.
- Contribute to sustainable health care initiatives, including carbon footprint reduction and integration of planetary health metrics into quality improvement.
- Collaborate with Indigenous communities, environmental NGOs, and public health agencies in One Health initiatives.

Further research priorities include evaluating the long-term health and ecological impacts of lifestyle medicine interventions that incorporate planetary health goals; understanding how to scale nature-based and green social prescribing programs; quantifying biodiversity outcomes of dietary and transport changes; and developing equitable strategies to ensure vulnerable populations benefit from co-designed interventions rather than bearing additional burdens.

## Conclusion

Species extinction and biodiversity loss are not distant environmental concerns but immediate determinants of human health and survival. Through mechanisms including altered infectious disease dynamics, compromised food security and nutrition, loss of medicinal resources and future solutions, climate destabilization, microbiome disruption, and increased metabolic and mental health burden, biodiversity decline threatens to undermine health going forward as a species.

Lifestyle medicine stands at a strategic crossroads. Its traditional focus on modifiable behaviours provides powerful levers to reduce the global burden of chronic disease. Yet the evidence reviewed here demonstrates that the same behaviours, dietary choices, transport modes, engagement with nature, patterns of consumption, also determine trajectories of biodiversity loss and planetary stability. A lifestyle medicine that remains silent on environmental implications will be increasingly misaligned with the realities of the Anthropocene.

Reframing lifestyle medicine through a planetary health and One Health lens reveals that health-promoting behaviours and ecosystem-protecting behaviours are often the same. Whole-food, plant-predominant diets reduce, inflammation and associated issues like metabolic and cardiovascular disease as well as free up land for restoration. Promoting active transport not only helps obesity and prevents type 2 diabetes, it also decreases habitat-fragmenting infrastructure and emissions. Nature-based stress management supports mental health and builds societal support for conservation. Socially connected, resilient communities cope better with climate-related disasters like floods famine and bushfires and can mobilize for biodiversity and forest protection from these very same disasters.

The central insight from evidence analyzed in this review is that there is no viable future for human health on a biologically impoverished planet. Lifestyle medicine must therefore evolve from a discipline primarily concerned with individual behaviour to one that recognizes and acts upon the deep entanglement of human health with the health of ecosystems. This entails integrating biodiversity and climate considerations into every existing pillar of lifestyle medicine practice, engaging in structural and policy advocacy, and partnering with other sectors and knowledge systems to build a just and equitable world on a safe planet; the only liveable planet we know of.

In this expanded paradigm, clinicians become not only partners and stewards of patient health but also advocates for the living systems that sustain all health and all life as we know it. The moral and scientific imperatives converge; protecting biodiversity is fundamentally an act of preventive medicine at planetary scale, and lifestyle medicine is uniquely positioned to help lead this reformation.

The Lancet One Health Commission’s call to action is that: *we cannot separate human health from the health of our planet*^1^ can be operationalized through lifestyle medicine’s evidence-based toolkit.

## Note

1. Dr Bob Brown, quote; as of 2^nd^ January 2026 https://en.wikipedia.org/wiki/Bob_Brown.
